# Emerging Smart and Adaptive Hydrogels for Next-Generation Tissue Engineering

**DOI:** 10.3390/bioengineering13010050

**Published:** 2025-12-31

**Authors:** Soheil Sojdeh, Amirhosein Panjipour, Miranda Castillo, Zohreh Arabpour, Ali R. Djalilian

**Affiliations:** Department of Ophthalmology and Visual Science, University of Illinois, Chicago, IL 60612, USA; sojdesoheil@gmail.com (S.S.); apanj@uic.edu (A.P.);

**Keywords:** tissue engineering, smart and adaptive biomaterials, responsive hydrogels

## Abstract

Tissue engineering is entering a new era, one defined not by passive scaffolds but by smart, adaptive biomaterials that can sense, think, and respond to their surroundings. These next-generation materials go beyond simply providing structure; they interact with cells and tissues in real time. Recent advances in mechanically responsive hydrogels and dynamic crosslinking have demonstrated how materials can adjust their stiffness, repair themselves, and transmit mechanical cues that directly influence cell behavior and tissue growth. Meanwhile, in vivo studies are demonstrating how engineered materials can harness the body’s own mechanical forces to activate natural repair programs without relying on growth factors or additional ligands, paving the way for minimally invasive, force-based therapies. The emergence of electroactive and conductive biomaterials has further expanded these capabilities, enabling two-way electrical communication with excitable tissues such as the heart and nerves, supporting more coordinated and mature tissue growth. Meanwhile, programmable bioinks and advanced bioprinting technologies now allow for precise spatial patterning of multiple materials and living cells. These printed constructs can adapt and regenerate after implantation, combining architectural stability with flexibility to respond to biological changes. This review brings together these cross-cutting advances, dynamic chemical design, mechanobiology-guided engineering, bioelectronic integration, and precision bio-fabrication to provide a comprehensive view of the path forward in this field. We discuss key challenges, including scalability, safety compliance, and real-time sensing validation, alongside emerging opportunities such as in situ stimulation, personalized electromechanical sites, and closed loop “living” implants. Taken together, these adaptive biomaterials represent a transformative step toward information-rich, self-aware scaffolds capable of guiding regeneration in patient-specific pathways, blurring the boundary between living tissue and engineered material.

## 1. Introduction

The remarkable progress in tissue engineering in the last few decades has attracted the attention of many scientists due to the urgent need to accelerate the healing of damaged tissues [1,2,3]. Primary biomaterial systems were only able to provide structural support for cells and could not be in direct contact with them due to their poor performance [4]. Artificial platforms that actively interact with biological tissues are the backbone of modern regenerative medicine [5]. In tissue engineering, materials science plays a crucial role, and selecting the right material is fundamental because the material must support biological function, guide regeneration, and ensure long-term compatibility [6].

Responsive hydrogels and other environmentally sensitive biomaterials have recently begun a period of profound changes in tissue engineering [7,8]. The extracellular matrix is a key component of all living organisms, and these stimuli-responsive hydrogels are three-dimensional networks of hydrophilic polymers designed to mimic it. Various physical, chemical, and biological stimuli play a crucial role as a trigger for smart biomaterials in biological systems for biomedical applications, including drug delivery, biosensor and tissue engineering [9]. It is possible to create microenvironments that enhance tissue healing by real-time regulation of edema, degradation, and drug/protein release. The fact that they are responsive makes this a real possibility [10].

**Scheme 1:**
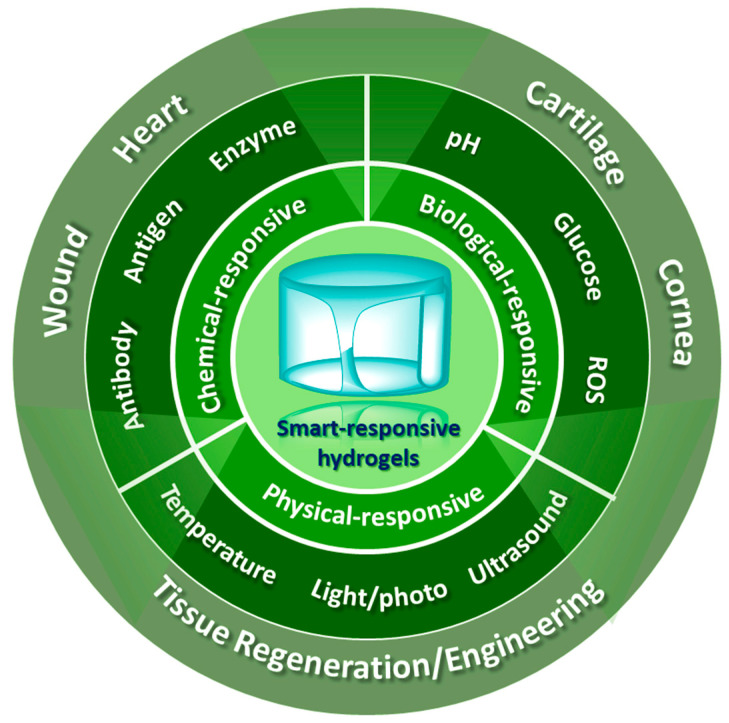
Classification and application of smart-responsive hydrogels in tissue regeneration/engineering.

In the field of tissue engineering, hydrogels are regarded as excellent biocompatible scaffold materials due to their network structure, high-water content, and tunable physicochemical properties, which closely mimic the native extracellular matrix [11,12]. Responsive hydrogels as smart and adaptive biomaterials have been demonstrated to facilitate the regeneration of damaged tissues by increasing cell proliferation, migration, and repairing effects [13]. The development of hydrogels has increased in recent years, with the advent of numerous smart-responsive hydrogel systems that have garnered significant attention. Smartly responsive hydrogels can respond to physical, chemical, and biological stimuli by altering their swelling or degradation behavior, thereby producing different textures [14,15]. This technique enables the regulated release of therapeutic agents, as well as the initiation of growth factor release and gene expression within engineered cells [16,17]. These processes subsequently promote cell proliferation and alter the surrounding microenvironment. Modifying their electrical properties, chemical composition, and stiffness allows one to tune these processes. Developing artificial tissues that are able to mimic the properties and activities of real tissues remains an elusive goal, despite significant advances in polymeric biomaterials. Recently, responsive smart hydrogels have attracted much attention due to their potential application in tissue regeneration. Compared with standard hydrogels, they are much more functional and flexible (Scheme 1).

The central motivation and discussion of this review lie in its systematic consideration and summation of the recent advances in stimuli-responsive hydrogels as smart and adaptive biomaterials for tissue engineering. This review aims to comprehensively overview the classification of smart-responsive hydrogels, understanding their stimulus response, exploring their potential applications in different tissues, and examining their properties are all areas that have been extensively studied. Integration of bioelectronics, real-time sensor validation, precision biomanufacturing, and mechanobiology-based design are all new research areas. The ultimate goal is to provide novel insights for the future design of more efficient and clinically translatable smart-responsive biomaterials for bone, cartilage, cornea regeneration, wound dressing, and next-generation tissue engineering.

## 2. Physical, Chemical, and Biological Responsive Hydrogels

In recent decades, a variety of hydrogels have been developed for tissue repair, serving as wound dressings, tissue adhesives, and carriers for cells, drugs, or proteins. Their 3D network structure and hydrophilicity provide a supportive environment for cell growth and enable targeted delivery of therapeutic agents. Smart-responsive hydrogels represent a significant advancement; they can sense and react to changes in their surroundings and offer advantages over traditional ones, including controlled delivery, improved effectiveness in tissue repairing, and reduced side effects. Classified as physical-, chemical-, or biological-responsive, smart hydrogels have attracted growing attention for their versatility and potential in regenerative medicine and bioengineering.

### 2.1. Physical Responsive Hydrogels

#### 2.1.1. Ultrasound-Responsive Hydrogels

As a new type of smart hydrogel, ultrasound-responsive hydrogels show increasingly powerful functions in bioengineering and biomedical applications due to their noninvasive, controllable, and safe properties. Ultrasound-responsive hydrogels have been used for designing stimuli-responsive drug delivery systems, biomedical imaging, smart materials, and tissue repairing [18,19]. For example, Zhang et al. [20] created a biomimetic peptide-based nanofibrous hydrogel that is activated by ultrasound and releases nanofibers when exposed to ultrasound waves. This hydrogel keeps tissue integrity, encourages M2-type macrophage polarization, and speeds up bone healing [20]. In another study, Chen et al. developed a battery-free vagal nerve stimulator utilizing programmable ultrasonic remote actuation and implanted high-performance hydrogel, successfully applying it in the anti-inflammatory treatment of sepsis. Using endotoxin-induced systemic inflammation as a model, ultrasound-responsive constant pressure pulse stimulation of vagal nerve stimulators significantly inhibited proinflammatory cytokines. The study proposed an innovative method for developing implantable soft nanogenerators for the radio stimulation of the nervous system [21]. As reported in another study, Zhao et al. developed an ultrasound-triggered tissue-penetrating hydrogel, which is capable of releasing thrombin through the action of ultrasound waves without invading the tissues, forming a fibrin hydrogel network, and promoting the reconstruction of the microvascular network [22]. In another investigation, a nanocomposite hydrogel was created by Xiao et al. and contains BST, gelatin, and organic carbon ions. This hydrogel stimulated bone tissue regeneration and showed a strong anticancer impact when exposed to a combination of near-infrared (NIR) and ultrasonic waves. Without undergoing invasive surgery to remove the tumor, this non-invasive therapy expedites bone mending while precisely treating malignancies [23].

The inherent properties of certain hydrogels, such as injectability, self-healing, and tunable adhesion, reflect the non-invasive characteristics of ultrasound and mitigate the substantial trauma linked to surgical procedures. For example, Zhou et al. developed a piezoelectric nanocomposite hydrogel that establishes dynamic covalent bonds, including Schiff bases and hydrogen bonds, enabling self-repair capabilities. Their macroscopic self-repair experiments included bisecting the hydrogel and then incubating the halves at 37 °C for a specified duration. Subsequently, they observed significant self-healing, and the halves effectively re-bonded. The extraordinary self-healing ability of the self-healed hydrogels was validated by dynamic mechanical analysis (DMA), revealing that their stress-strain curves closely resembled those of the original samples [24].

Also, Zhou et al. developed a hydrogel with tunable adhesion by including barium titanate nanoparticles through amino acid substitution. The system’s adhesive capacity is markedly improved by the interaction of inorganic nanoparticles with the polymeric hydrogel framework, which fortifies the link between the bone surface and the matrix. The nanocomposite hydrogel generated a controlled electrical output under ultrasonic radiation, enhancing the efficacy of bone attachment. We assessed the adhesion properties of the hydrogels to bone tissue by in vitro adhesion experiments on newly obtained pig femurs [24].

Ultrasound-responsive hydrogels constitute a versatile class of smart biomaterials that exploit the non-invasive and remotely controllable nature of ultrasound while achieving distinct biological functions through tailored material design. Comparative analysis of recent studies reveals that peptide-based and fibrillar hydrogels primarily rely on ultrasound-triggered structural or molecular release to modulate immune responses and promote tissue regeneration, whereas nanocomposite and piezoelectric hydrogels integrate electroactive components to enable neuromodulation, enhanced bone adhesion, or synergistic anticancer and regenerative effects. These differences highlight a key trade-off between simplicity and multifunctionality, with increasing compositional complexity offering broader therapeutic outcomes at the cost of system design and translational challenges. Common design principles such as injectability, self-healing, and tunable adhesion align closely with the non-invasive stimulus modality, yet unresolved issues remain regarding long-term stability, precise signal control, and clinical scalability.

#### 2.1.2. Magnetic-Responsive Hydrogels

The integration of magnetic nanoparticles into hydrogels facilitated their design and synthesis; these hydrogels have significant potential for tissue regeneration due to their noninvasive remote activation, rapid magnetic responsiveness, and accurate spatiotemporal regulation [25]. Magnetic nanomaterials include iron oxides (Fe_3_O_4_, γ-Fe_2_O_3_), transition metal ferrites (CoFe_2_O_4_, MnFe_2_O_4_, etc.), and transition metal alloys (FePt) [26,27]. The biocompatibility, high magnetic susceptibility, relatively simple production, and functionalization of Fe_3_O_4_ nanoparticles make them a favored option among magnetic nanomaterials [28]. The depth of tissue penetration for treating produced responsive hydrogels, which exhibit various response modes such as motion, deformation, and heat generation, is unlimited when influenced by an external magnetic field [29]. Composite materials, including magnetically sensitive hydrogels, may exhibit properties contingent upon many factors. Numerous variables influence the physicochemical properties of magnetic responsive hydrogels, including the network structure of the hydrogels and the kind, content, size, and distribution of the magnetic nanoparticles [30].

Attaining the required characteristics in magnetic-responsive hydrogels depends on the meticulous selection of hydrogel constituents and magnetic nanoparticles. For instance, Kondaveeti et al. revealed magnetic-responsive hydrogels based on alginate and xanthan gum with magnetic nanoparticles. The obtained results demonstrated that the magnetic alginate-xanthan hydrogels have high electric potential and superior mechanical properties. The fabricated magnetic-responsive hydrogel could promote cell proliferation and specific differentiation under the stimulation of the external magnetic field [31]. As hydroxyapatite (HAP) is one of the most important components in natural bone inorganic substances, exhibits excellent biocompatibility and osteo-conductivity, and plays a key role in biomineralization, the magnetic HAP composite hydrogel was designed. For example, Iqbal et al. synthesized coated nano-HAP with γ-Fe_2_O_3_ nanoparticles (m-nHAP) and then added them into poly (vinyl alcohol) (PVA) solution to fabricate m-nHAP/PVA hydrogels. The PVA showed excellent biocompatibility, high mechanical properties, and slow biodegradation, which were crucial for bone tissue engineering. The pore sizes of hydrogels rose gradually, followed by the increased content of m-nHAP, which was accessible for nutrient exchange [32].

Recent endeavors of magnetic hydrogels have been developed to deal with Cartilage injury as one of the most common types of orthopedic disease. Zhang et al. modified PVA with Fe_3_O_4_ magnetic nanoparticles and then mixed it with a hybrid hydrogel composed of hyaluronic acid, type II collagen, and polyethylene glycol by mechanical dispersion. After in vitro hydrogel degradation, the magnetic nanocomposite hydrogel showed a similar microstructure and chemical components in comparison with natural hyaline cartilage and was able to support bone mesenchymal stem cells’ behavior [33]. The stem cell therapy has been introduced into the treatment of cartilage injury [34]. In another considerable study on magnetic-responsive hydrogels in tissue engineering, researchers could promote neural regeneration directly via designing magnetic-responsive hydrogels [35]. For instance, the novel “Anisogel” was created by Omidinia-Anarkoli et al. utilizing a high-throughput electrospinning/micro cutting technology and short, magnetically-inspired poly(lactide-co-glycolide) (PLGA) fibers. In contrast to cases without fibers or with just oriented fibers, their results showed that nerve cells attached well and expanded in a unidirectional way inside the Anisogel. Furthermore, neurons embedded in the aligned magnetic hydrogel exhibited calcium signal-dependent spontaneous electrical activity. Next, they used electrospinning to create magnetic poly-L-lactic acid (PLLA) fibers, which they then incorporated into a hydrogel solution containing collagen or fibrinogen. Magnetic nanoparticles (MNPs) were oriented inside these PLLA fibers by applying an external magnetic field. The alignment greatly enhanced the direction guidance of neurons, leading to longer neurite extensions on magnetic fibers from dorsal root ganglions (DRGs) as compared to neurons without MNP inclusion [36].

Besides the bone, cartilage, and nerve organs, magnetic hydrogels are also introduced into other organs, such as the heart, skin, and muscle, in order to evaluate the therapeutic potential. Namdari et al. designed a magnetic hydrogel by dissolving Fe_3_O_4_ and curcumin into the N-isopropylacrylamide-methacrylic acid hydrogel. The resulting magnetic hydrogel nanocomposite was able to reduce the doxorubicin-induced cardiac toxicity and hold the cardioprotective capability [37].

Magnetic-responsive hydrogels exemplify how a common actuation modality—external magnetic fields—can be translated into diverse regenerative outcomes through compositional and structural tuning. While iron oxide–based systems, particularly Fe_3_O_4_ nanoparticles, are favored for their biocompatibility and ease of functionalization, their integration into different polymeric networks yields distinct mechanistic advantages, ranging from magnetically induced motion and deformation to localized heating and electrical stimulation. Comparative evaluation of recent studies shows that alginate-, PVA-, and ECM-inspired hydrogels primarily target bone and cartilage regeneration by enhancing mechanical integrity, porosity, and stem cell differentiation, whereas anisotropic, fiber-reinforced magnetic hydrogels introduce directional cues that are critical for neural regeneration. These contrasts highlight a key design trade-off between isotropic bulk reinforcement for load-bearing tissues and anisotropic architectures for guidance-dependent tissues such as nerves. Beyond musculoskeletal and neural applications, magnetic hydrogels have also demonstrated cardioprotective and anti-inflammatory functions, underscoring their multifunctional potential. Nonetheless, challenges remain in precisely controlling nanoparticle distribution, long-term magnetic stability, and biosafety, which must be addressed to enable reliable clinical translation.

#### 2.1.3. Photo-Responsive Hydrogels

Recent years have seen light/photo as a viable trigger because of its noninvasiveness, great spatiotemporal resolution, and low pollution [38]. Near-infrared, visible, and ultraviolet light are used. Light-responsive hydrogels may translate optical impulses into numerous functionalities, making them popular [39]. Light-responsive hydrogels change in three ways [40]. The major photo response mechanism in hydrogels is phase transition, triggered by photon absorption at specified energy levels by photosensitive group grafting. Azobenzene, diaryne, spiropyrans, photo-reversible dimerization groups (coumarin, anthracene, and pyrimidine derivatives), and nitrobenzyl derivatives are common photoactive groups [41]. The second light-responsive approach includes hydrogels containing photoactive chemicals that react with the network structure or osmotic pressure to generate swelling [42]. Third, photosensitive hydrogels absorb photon energy to modify their characteristics in response to environmental changes [43]. There has been wide application of light as a simple, nonintrusive, precise, and controllable stimulus in modern medicine, especially in tissue engineering [44].

Recently, Tang et al. cosprayed poly-3-hexylthiophene nanoparticles together with collagen and polycaprolactone (PCL) to construct a photo-guided bioactive scaffold (PCL-P3HT-Col), and fulfilled the neuronal differentiation with a remote and radiation stimulation [45]. The multitude of anatomical and physiological factors makes the treatment of central nervous system disorders more complex than that of peripheral nerve injuries. The recent creation of a PA hydrogel scaffold has rendered percutaneous wire attachment and viral transfection unnecessary for the effective enhancement of neurosynaptic growth [46]. This hydrogel incorporates PEG-functionalized carbon nanotubes (CNTs) to convert pulsed near-infrared light (NIR-II) into acoustic waves, serving as a photoacoustic (PA) agent [47]. An acoustic wave exhibiting a wide frequency spectrum was generated by rapid Fourier transform analysis, utilizing a 200 μm diameter multimodal fiber to convey 1030 nm laser light over a 0.05 mm^2^ area. Pulsed laser-illuminated neurons exhibited enhanced calcium influx, thereby initiating many regeneration processes, including the expression of neurotrophic factors such as BDNF, facilitated by the acoustic waves created by NIR-II [47].

Bacteria that are resistant to drugs, such as methicillin-resistant Staphylococcus aureus (MRSA), make infections, which are chronic wounds, very difficult to treat. The use of free radicals, such as singlet oxygen, is an efficient method of disinfection. This is an area where photo-responsivity hydrogels show great promise [48]. For instance, selenoviologen (SeV^2+^)-conjugated polythiophene (SeV^2+^-PT) is a dual-responsive wound dressing that has been created inside polyacrylamide hydrogel [49]. This treatment retains polythiophene’s photochromic and electrochromic characteristics while responding to both visible light and electricity, making liquefaction easier. The production of selenoviologen radical species is ensured by this process, which involves intramolecular electron-transfer driven by white light or electrification. The dual stimulation successfully reduces MRSA colony-forming units (CFU) by 98.4 percent by stimulating radicals and increasing the fluorescence of reactive oxygen species (ROS). When applied for seven days in conjunction with both light and electrical stimulation, the SeV^2+^-PT dressing facilitated the most rapid recovery from skin injuries [49].

In another study, to combat bacteria and promote wound healing, Yang et al. created a unique hydrogel that responds to near-infrared light. This multipurpose dressing is very adaptable, has great tissue adherence, and is easy to inject. The hydrogel’s photothermal agent generates a great deal of heat when exposed to near-infrared light, which allows for the localized release of antibiotics to eradicate bacteria from the wound [50]. A multifunctional hydrogel was also developed by Kuang et al. This gel contains parathyroid hormone and calcium phosphate nanoparticles; when exposed to near-infrared light, it may be activated to promote bone repair. The results showed that this hydrogel improves calvarial abnormalities and increases osteoblast and osteoclast activity, making it a potential therapy option for osteoporosis [51].

Light-responsive hydrogels exemplify how a single noninvasive stimulus can be harnessed through distinct photochemical and photophysical mechanisms to address diverse biomedical challenges. While most systems rely on photon-induced phase transitions mediated by grafted photoactive groups, others exploit light-driven swelling, photothermal conversion, or photoacoustic transduction, underscoring the versatility of optical triggers. Comparative analysis of recent studies reveals that photoresponsive scaffolds incorporating conductive polymers or carbon-based nanomaterials primarily target neural regeneration by enabling remote, wire-free stimulation and enhanced neurotrophic signaling, whereas photothermal and radical-generating hydrogels are optimized for antimicrobial wound healing through localized heat or reactive oxygen species production. In contrast, NIR-activated composite hydrogels loaded with bioactive agents emphasize controlled release and tissue-specific regeneration, such as bone repair. These examples highlight a key trade-off between precise spatiotemporal control and penetration depth, particularly when comparing UV/visible and NIR modalities, while also pointing to ongoing challenges in balancing phototoxicity, efficiency, and long-term safety for clinical translation.

#### 2.1.4. Thermo-Responsive Hydrogels

Thermoresponsive hydrogels consist of a combination of hydrophilic and hydrophobic polymers, enabling them to experience sol-gel phase transitions [52]. They are formed by hydrophobic interactions [53]. The absence of detrimental cross-linking agents, along with the ability of these hydrogels to solidify at elevated temperatures and revert to a liquid state at reduced temperatures, enhances their biocompatibility [54]. A significant biological application that notably benefits from their ability to transition at physiological temperatures is the creation of in situ hydrogels at body temperature for injections [55]. Lee et al. developed a thermosensitive hydrogel using the K9-C peptide, which demonstrated prolonged protection against inflammation and oxidative damage in animal models. This renders it a suitable option for prolonged use in individuals with diabetic vascular dysfunction, a condition associated with diabetes mellitus [56]. Moreover, these hydrogels function as a barrier, preventing tendon adhesions; for instance, Chou et al. demonstrated that a poly-n-isopropyl acrylamide-based hydrogel efficiently inhibited postoperative peritendon adhesions [57]. Furthermore, Yu et al. enhanced the effectiveness of conventional wound care techniques by developing adhesive heat-contracting hydrogels with optimal mechanical properties; these hydrogels facilitated rapid incision closure due to robust tissue adhesion and thermal contraction [58].

Dental caries is a significant health issue that leads to demineralization and several adverse effects. Licorice (LC) is a potent natural isoflavonoid beneficial for tooth health due to its germicidal properties. Nevertheless, it is rapidly decomposed and has poor solubility in water. Li et al. developed an ingestible temperature-sensitive hydrogel (CS/ŗ-GP) to enhance the oral delivery of LC. This hydrogel effectively combats bacterial biofilms and facilitates the gradual release of medications [59]. A thermally sensitive CS/gelatin/glycerol phosphate hydrogel injected with induced pluripotent stem cells (iPSCs) and bone morphogenic protein-6 (BMP-6) has been shown to enhance mineralization in maxillary-molar abnormalities by augmenting bone thickness and trabecular density [60]. The integration of a β-TCP-β-GP-chitosan/collagen hydrogel with the polyphenolic chemical quercetin led to improved sustained release and osteoconductive characteristics, promoting cell proliferation and osteoblast differentiation [61]. Numerous studies have shown the significance of thermosensitive hydrogels such as CS/HAP/Hep in enhancing microvascularity and angiogenesis during bone regeneration [62]. Tian et al. developed a thermo-responsive hydrogel including Levo-DOPA for the treatment of bacterial lesions in rats [63].

Castillo-Henríquez et al. said that a CS/PVA/gelatin hydrogel is capable of gradually releasing dexketoprofen trometamol [64]. Headaches and other symptoms may exacerbate temporomandibular joint (TMD) disorders [65]. When administered directly into a joint, temperature-sensitive hydrogels transition from a liquid to a solid state, facilitating the proliferation of chondrocytes and the synthesis of glycosaminoglycans 54. In this regard, ElTahir et al. developed graphene oxide (GO) to CS/β-GP to create an injectable thermosensitive hydrogel loaded with bupivacaine hydrochloride (BH) release. In a concentration-dependent way, the addition of GO greatly enhanced the hydrogel scaffolds’ biomechanical characteristics. Studies conducted in vivo showed that this platform greatly extended the local anesthetic effect and increased the blockage of the pain sensory reflex [66].

A temperature-sensitive peptide hydrogel adorned with IKVAV peptide was created by Chai et al. It exhibited a regular 3D porous structure, great biological activity, and quick (de)swelling capabilities. The scientists demonstrated that this scaffold increased angiogenesis, inhibited keratinocyte differentiation and adhesion, and reduced glial scar tissue production after using it to treat spinal cord damage. They proved that this hydrogel works well by increasing blood vessel formation and decreasing cytokine production that promotes inflammation. The biomaterial’s ability to inhibit the formation of glial scar tissue was crucial in facilitating the repair of injured tissues [67,68].

Furthermore, the bi-layer photo-thermal-responsive hydrogel nanocomposites created by Radojković et al. include Poly(N-isopropylacrylamide) with an active layer of AuNPs and a passive layer of PVA. Thermoresponsive PNiPAAm and photothermal AuNPs work together in a complementary manner because of this. There are clear advantages to this technology, and one of them is the use of gamma irradiation to create bi-layered hydrogel nanocomposites in a sustainable and environmentally responsible way. Soft robots, medical devices, and responsive materials are all areas where this method might be useful [69].

Thermoresponsive hydrogels illustrate how intrinsic polymer chemistry can be harnessed to achieve temperature-dependent sol-gel transitions, enabling minimally invasive delivery and in situ formation at physiological temperatures. Comparative analysis of recent studies shows that simple thermosensitive systems, such as poly-N-isopropyl acrylamide or chitosan/gelatin hydrogels, are primarily used for localized drug delivery, wound healing, and prevention of tissue adhesions by exploiting thermal gelation and adhesive properties. In contrast, multifunctional thermoresponsive composites incorporate bioactive molecules, nanoparticles, or photothermal agents to combine sustained drug release, osteoconductivity, enhanced mechanical performance, or dual responsiveness to light and temperature, thereby expanding applications to bone regeneration, dental therapy, pain management, and soft robotics. These examples highlight a balance between biocompatibility and functional complexity: while simpler hydrogels ensure safe and effective local administration, composite systems enable tailored mechanical, biochemical, and stimuli-responsive properties, although they require careful optimization for translational feasibility.

#### 2.1.5. Pressure-Responsive Hydrogels

Pressure-responsive hydrogels sensitive to variations in external pressure may undergo structural phase transitions [70]. They react variably to many stressors and display a broad spectrum of behaviors, consequently [71]. Pressure-responsive hydrogels, such as alginate, flexible SiO_2_ nanofiber, cellular, and nanofibrous hydrogels, may be synthesized in a water-rich environment [72,73]. Despite the uniform distribution of these hydrogels’ networks, their mechanical characteristics often deteriorate in regions with high water content. There are various research papers on designing pressure-responsive hydrogels for next-generation tissue engineering. For instance, Di et al. proposed a wearable medication delivery system that integrates microgels with flexible elastomers to encapsulate drug-loaded nanoparticles. The drug release from microdepots under tensile strain was enhanced due to the augmented surface area for compression and diffusion, as elucidated by Poisson’s ratio [74]. In another study, Korin et al. created a drug delivery device that selectively distributes medications at regions of vascular stenosis when exposed to severe shear stress. This design concept centers on microaggregates of shear-sensitive nanoparticles that adhere to the interiors of constricted blood arteries and disintegrate into their constituent nanoscale components in regions of elevated fluid shear stress. This innovative therapy approach may provide optimism to patients experiencing hemodynamic challenges due to pulmonary embolism, stroke, or atherosclerosis [75]. In another investigation, Zhu et al. used a monomer polymerization approach to construct a hydrogel sensor with a semi-interpenetrating network, yielding a distinctive stretchy piezoresistive strain sensor. The ability to sense minute vibrations and human movements is facilitated by the remarkable mechanical characteristics and recoverability of hydrogel strain sensors with a high gauge factor. An efficient and accessible method for managing medication distribution included using a stimulus derived from mechanical force [76].

Pressure-responsive hydrogels demonstrate how mechanical stimuli can be translated into functional material responses through structural phase transitions and network design. Comparative evaluation of recent studies shows that simple pressure-sensitive hydrogels, such as alginate or nanofibrous systems, primarily exploit changes in network compression and porosity to modulate drug release or mechanical behavior, whereas more sophisticated designs integrate microgels, shear-sensitive nanoparticles, or semi-interpenetrating polymer networks to achieve targeted delivery or high-fidelity strain sensing. For example, wearable drug delivery platforms leverage tensile strain to enhance nanoparticle diffusion, while vascular-targeted systems release therapeutics selectively at stenotic regions under high shear stress. Similarly, piezoresistive hydrogel sensors convert minute mechanical deformations into detectable electrical signals, highlighting their potential for real-time monitoring of physiological movements. These studies collectively illustrate the trade-offs between material simplicity, responsiveness, and functional precision, emphasizing the importance of network architecture, mechanical properties, and stimulus sensitivity in designing pressure-responsive hydrogels for tissue engineering and biomedical applications.

### 2.2. Chemical Responsive Hydrogels

#### 2.2.1. ROS-Responsive Hydrogels

Reactive oxygen species (ROS) are crucial for maintaining cellular homeostasis; yet, an excess of these molecules in damaged tissues can result in many illnesses [77]. Consequently, employing biological products as catalysts to manipulate and regulate the structure and function of polymers is a potentially effective therapeutic strategy. In a high ROS environment, the architecture of ROS-responsive hydrogels may be altered, modulating drug release while minimizing adverse effects on healthy cells [78]. A growing variety of clinical illnesses are associated with heightened levels of ROS. The study area of ROS-responsive hydrogels has markedly expanded. Research indicates that spinal cord injury is a degenerative phenomenon characterized by complex inflammation. The excessive accumulation of ROS during this inflammation might result in the demise of neuronal and glial cells [79].

For example, Zhao et al. successfully synthesized a polyvinyl alcohol (PVA) hydrogel that efficiently scavenges ROS by cross-linking with a ROS-responsive linker. The hydrogel facilitated wound healing by increasing M2 phenotype macrophages and decreasing ROS levels [80]. In addition, Li et al. elaborated on the methodology for fabricating a multifunctional hydrogel coating that binds firmly to titanium implants infused with Tβ4 and responds to ROS. This coating aims to enhance osseous development and vascular repair through immunomodulation [81]. In another study, Zheng et al. created a ROS-responsive PAMB-G-TK/4-arm PEG-SG hydrogel for the injection of tailored drug-encapsulated liposomes. This hydrogel enhances cardiomyocyte function and ameliorates myocardial infarction therapy by absorbing excessive ROS [82]. As another witness for applicability of ROS-responsive hydrogels for tissue engineering, Li et al. formulated a thioketone-infused hydrogel that captures ROS to encapsulate bone marrow mesenchymal stem cells. The ROS-scavenging hydrogel enhanced spinal cord tissue regeneration by mitigating ROS-mediated oxidative damage and downregulating inflammatory cytokine production. ROS generated by injuries or bacterial infections are a principal impediment to wound healing [83].

ROS-responsive hydrogels exemplify the therapeutic potential of designing materials that actively interact with the biochemical microenvironment, converting pathological oxidative stress into controlled functional outcomes. Comparative analysis of recent studies shows that simple ROS-scavenging hydrogels, such as PVA cross-linked with ROS-sensitive linkers, primarily reduce oxidative damage and modulate immune responses to promote wound healing, whereas multifunctional systems incorporate bioactive molecules, drug-loaded liposomes, or stem cells to simultaneously regulate inflammation, enhance tissue regeneration, and support vascular or osseous repair. For instance, ROS-responsive coatings on titanium implants facilitate immunomodulation and bone growth, while thioketone-infused hydrogels protect spinal cord tissue by mitigating ROS-mediated neuronal damage. These approaches highlight a trade-off between straightforward antioxidant activity and multifunctional tissue-targeted therapies, emphasizing that careful material design, including polymer composition, crosslinker choice, and cargo integration, is essential to balance ROS scavenging, biocompatibility, and regenerative efficacy.

#### 2.2.2. pH-Responsive Hydrogels

The physical and chemical characteristics of pH-responsive hydrogels may alter within a given pH range [84]. Protonation happens at lower pH levels, while deprotonation occurs at higher pH values for the polymer chain’s acidic groups [85]. The changes that occurred in the structure of the hydrogel were brought about by the interaction and dissociation of a number of different ions with the polymer chain 60. Since various chronic ailments, such as tumors and wounds, commonly have mildly acidic surroundings, a variety of pH-responsive hydrogels have been created lately to treat these conditions [86].

A pH-responsive injectable multifunctional hydrogel was designed by Hu et al. based on the moderately acidic environment of wounds that are chronically infected. In order to successfully remove germs at chronic wound sites, pH-responsive hydrogels swiftly released silver nanoparticles. Additionally, the medication deferoxamine encouraged the development of new blood vessels, which hastened the healing process of chronic skin wounds [87].

Utilizing glucono δ-lactone as an acidifier inside a carboxymethyl chitosan/amorphous calcium phosphate (CMC-ACP) hydrogel system, Chen Zhao and colleagues have successfully developed a hydrogel that is sensitive to changes in pH and may be subcutaneously administered. The expression of bone markers such as Runx2, osterix, osteocalcin, and osteopontin was greatly boosted by the hydrogel system. This was in addition to the fact that the hydrogel system promoted the proliferation of mesenchymal stem cells, biocompatibility, osteoinduction, and strong cell adhesion. The results from the in vivo study indicate that the injectable hydrogel containing CMC-ACP-BMP9 blocks the process of bone resorption while simultaneously significantly boosting the efficiency and maturation of bone regeneration [88].

pH-responsive hydrogels illustrate how environmental acidity can be leveraged to achieve targeted and controlled therapeutic effects. Comparative evaluation of recent studies shows that simpler systems, such as silver nanoparticle–loaded hydrogels, exploit acidic wound microenvironments to trigger rapid antimicrobial release and promote angiogenesis, whereas more complex composites, like carboxymethyl chitosan/amorphous calcium phosphate hydrogels, integrate bioactive molecules such as BMP9 to simultaneously enhance stem cell proliferation, osteogenic differentiation, and bone regeneration. These examples highlight a common design principle: protonation–deprotonation dynamics enable site-specific responsiveness, but the choice of polymer matrix, incorporated therapeutics, and acidifiers determines the balance between antimicrobial, regenerative, and osteoinductive functionalities. Consequently, pH-sensitive hydrogels provide a versatile platform for localized treatment of wounds, bone defects, and other mildly acidic pathological sites, with tunable properties tailored to specific clinical needs.

#### 2.2.3. Glucose-Responsive Hydrogels

Different amounts of glucose can make glucose-responsive hydrogels react [89]. There are two great uses for glucose-responsive hydrogels: blood glucose sensors and medicine delivery systems that work on their own [90]. The main ways to classify them are glucose oxidase (GOx), concanavalin A (Con A), and phenylboronic acid (PBA). One of these enzymes, GOx, speeds up the oxidation of glucose. However, H_2_O_2_, a by-product, causes inflammation. The lectin Con A has four glucose-binding sites, even though it is very toxic to cells. PBA is a very stable synthetic compound. PBA is better than GOx and Con A when it comes to ease of use and cost. Currently, there is no effective treatment for persistent wounds related to diabetes [91,92].

Xu et al. created a new glucose-responsive hydrogel by combining polyethylene glycol diacrylate with PBA that had been modified onto a hyaluronic acid chain. When glucose is present, the multifunctional hydrogel may be able to get rid of reactive oxygen species and help release myricetin, an antioxidant, in a controlled way. Glucose-responsive hydrogels can help heal bones and cartilage. These hydrogels might control the microenvironment by releasing bioactive molecules like ECM proteins and growth factors when the glucose level changes. This would make osteoblasts stick to each other, multiply, and change into different types of cells 104. Liu et al. used dynamic borate bonding to attach PBA to the GelMA chain to make resveratrol (Res) stay in place. Res is let go when borate bonds break down in conditions of high blood sugar. Res improves the environment for bone repair, encourages the growth and mineralization of osteoblasts, and has anti-inflammatory and antioxidant properties. At the same time, models of bone defects may use glucose-responsive hydrogels that are carefully combined with bone tissue to make a strong interface that keeps nearby tissues from interfering. Also, hydrogels with a three-dimensional network structure may help drugs get to where they need to go and act as a stabilizing medium for 3D-printed scaffolds [93,94].

In a different study, Zhao et al. made hydrogels that change when the pH changes or when glucose is present. They made injectable hydrogels that respond to pH and glucose by using phenylboronic modified chitosan, PVA, and benzaldehyde-capped poly (ethylene glycol). We did this by using Schiff’s base and phenylboronate ester reactions. During the in-situ crosslinking process, hydrogels can incorporate protein therapeutics and viable cells. Because they can release drugs when the pH or glucose levels are high, and because they can keep cells alive and help them grow inside the 3D matrix, the hydrogels could be used as bioactive dressings for diabetic wounds [95].

Glucose-responsive hydrogels exemplify how biochemical stimuli can be harnessed for dynamic therapeutic control, particularly in diabetes-related applications. Comparative analysis shows that enzyme-based systems, such as glucose oxidase (GOx), and lectin-based systems, like Con A, provide glucose sensitivity but suffer from cytotoxicity or inflammatory by-products, whereas phenylboronic acid (PBA)-based hydrogels offer greater stability, biocompatibility, and cost-effectiveness. PBA-functionalized hydrogels have been effectively integrated with bioactive molecules such as myricetin or resveratrol to achieve controlled antioxidant release, modulate the cellular microenvironment, and enhance osteoblast proliferation, differentiation, and bone repair. Furthermore, multifunctional hydrogels that combine glucose and pH responsiveness enable in situ encapsulation of therapeutics and viable cells, providing bioactive scaffolds for wound healing or tissue engineering. These studies underscore a trade-off between stimulus specificity, biocompatibility, and multifunctionality, highlighting how polymer chemistry and network architecture dictate the therapeutic precision and regenerative potential of glucose-responsive hydrogels.

### 2.3. Biological Responsive Hydrogels

#### 2.3.1. Enzyme-Responsive Hydrogels

Enzyme-responsive hydrogels are distinctive intelligent materials. The structure of hydrogel undergoes alterations due to selective enzyme catalysis. Recent advancements in bioengineering indicate that enzymes may enhance the reactivity of hydrogels [96,97].

Skaalure et al. used chaldrocyte activity to develop a cartilage-specific, enzymatically responsive PEG hydrogel. Cartilage mostly consists of elastic collagen II fibrils and proteoglycans, predominantly aggrecan, interspersed with a limited number of chondrocytes that modify the extracellular matrix. They subsequently developed a peptide sensitive to aggrecanase that specifically targets an IGD cleavage site. Human and bovine aggrecan have the identical amino acid sequence TEGE-ARGSVI at the E373-A374 cleavage site. To enhance solubility, ‘RD’ moieties were included, and thiol-containing cysteines were introduced for crosslinking, yielding CRDTEGE-ARGSVIDRC. A crosslinker was incorporated into a photo-clickable thiol-ene PEG hydrogel with a precisely specified network structure to produce a novel aggrecanase-sensitive hydrogel. This bovine chondrocyte source, exhibiting unique anabolic and catabolic properties, was utilized to evaluate this novel hydrogel for cartilage tissue engineering [98].

Gliomas are the most aggressive primary malignant brain tumors. Temozolomide (TMZ) is used for the treatment of gliomas post-surgery; nevertheless, its efficacy is constrained. Zhao et al. developed an injectable hydrogel laden with drugs that reacts to matrix metalloproteinase (MMP) enzymes to eliminate drug-resistant gliomas post-surgery. Post-glioma surgery, drugs were administered owing to elevated MMP enzymes, significantly enhancing TMZ’s efficacy in diminishing glioma proliferation [99].

Cai et al. synthesized MMP-2 degradable hydrogels by crosslinking allyl glycidyl ether grafted carboxymethyl chitosan with the peptide substrate CPLGLAGC. The combination of TGF-β1 siRNA inhibited fibroblast proliferation. In an in vivo setting characterized by MMP-2 overexpression, hydrogels delivered TGF-β1 siRNA to enhance peritendinous anti-adhesion [100].

Enzyme-responsive hydrogels represent a class of intelligent materials that exploit selective enzymatic activity to trigger structural and functional changes, enabling precise, site-specific therapeutic interventions. Comparative analysis of recent studies shows that cartilage-targeted hydrogels, such as aggrecanase-sensitive PEG systems, leverage tissue-specific enzyme recognition to remodel the extracellular matrix and support chondrocyte function, whereas MMP-responsive hydrogels are designed for pathological environments, including post-surgical gliomas or tendon injury sites, to achieve localized drug release or gene silencing. These systems demonstrate a trade-off between specificity and versatility: peptide- or substrate-based crosslinkers confer highly selective responsiveness, yet require careful design to match enzyme expression profiles, while broader enzyme-sensitive platforms allow multifunctional delivery but may risk off-target degradation. Overall, enzyme-responsive hydrogels highlight the potential of integrating biochemical cues with material architecture to achieve controlled, disease- or tissue-specific therapeutic outcomes.

#### 2.3.2. Antigen/Antibody-Responsive Hydrogels

The fundamental mechanism of antigen/antibody-responsive hydrogels operates through the specific binding response between the two entities [101]. To fabricate hydrogels that react to both antigens and antibodies, one may either include antigens directly into the hydrogels or chemically conjugate or copolymerize the binding fragments of the antigens with the hydrogels [102].

Choi et al. proposed a cost-effective and straightforward label-free detection technique for immunoglobulin G antibodies. The three-dimensional hydrogel structure revealed advantages such as a high signal-to-noise ratio, sensitivity, and superior immobilization capacity of biomolecules, hence increasing the probability of interactions with target ligands. Furthermore, antigen-responsive hydrogels have several potential applications, including drug delivery systems and antigen-detection sensors [103]. In another study, Yang et al. developed an aptamer-crosslinked hydrogel for the visual and quantitative detection of ochratoxin A. Hydrogels were synthesized by hybridizing complementary DNA strands grafted onto polyacrylamide with DNA strands containing ochratoxin A aptamers. A new method for ochratoxin A detection was developed by combining sensitive hydrogels with a portable enrichment technique [104].

Antigen/antibody-responsive hydrogels exemplify intelligent materials that harness highly specific molecular recognition to achieve targeted sensing and delivery functions. Comparative evaluation of recent studies indicates that simpler systems, in which antigens or antibody fragments are directly incorporated into hydrogels, primarily serve as platforms for label-free detection with high sensitivity and immobilization capacity, as demonstrated in immunoglobulin G assays. In contrast, more sophisticated designs, such as aptamer-crosslinked hydrogels, enable both visual and quantitative detection of specific targets like ochratoxin A, often integrated with portable enrichment techniques for enhanced practicality. These examples highlight a trade-off between structural simplicity and functional sophistication: while direct incorporation ensures straightforward fabrication and rapid responses, hybridized or aptamer-based networks provide improved selectivity, signal amplification, and multifunctionality, broadening the potential applications of antigen/antibody-responsive hydrogels in biosensing and controlled drug delivery.

Stimulus-responsive hydrogels represent a versatile class of smart biomaterials whose properties can be finely tuned by physical, chemical, or biological cues. As summarized in Table 1, each hydrogel type operates via a distinct mechanism, from acoustic or magnetic actuation to enzyme-mediated cleavage or antigen–antibody recognition, enabling precise control over swelling, degradation, or payload release. Physical stimuli such as ultrasound, light, temperature, and pressure allow non-invasive and spatiotemporally regulated responses, whereas chemical triggers like pH, glucose, or reactive oxygen species provide site-specific and self-regulated functionality. Biological cues, including enzymes and antigen–antibody interactions, offer tissue-specific responsiveness and high selectivity. The advantages of these systems, ranging from injectability, self-healing, and deep tissue penetration to high specificity and multifunctionality, make them suitable for a wide spectrum of applications, including tissue regeneration, targeted drug delivery, wound healing, neural and bone repair, and biosensing. This comparative framework underscores the complementary strengths of different hydrogel classes and provides guidance for their rational design in advanced biomedical applications.

## 3. Mechanobiology-Informed Adaptive Scaffolds

**Scheme 2:**
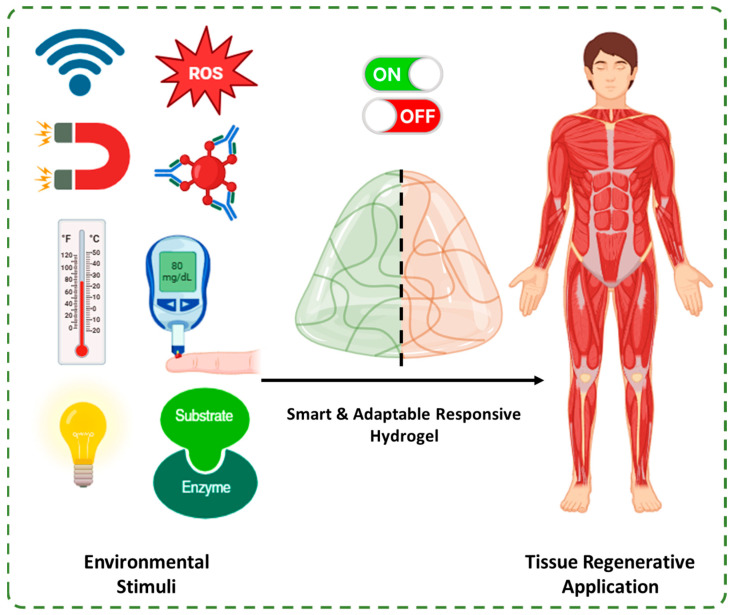
Schematic of physical, chemical, and biological stimuli effect on responsive hydrogels for tissue regenerative applications.

Cells exist in a constantly changing mechanical environment. They do not passively occupy space within a scaffold or tissue; they actively push, pull, and sense the stiffness and resistance of their surroundings. Through these interactions, they convert mechanical responses from their environment into biochemical messages that shape their behavior, including how they spread, migrate, proliferate, or differentiate (Scheme 2). This process, known as mechanotransduction, is highly sensitive not just to how stiff a material is, but also to how that stiffness changes over time [105].

### 3.1. Mechanical Foundations of Mechanosensitive Materials

**Figure 1:**
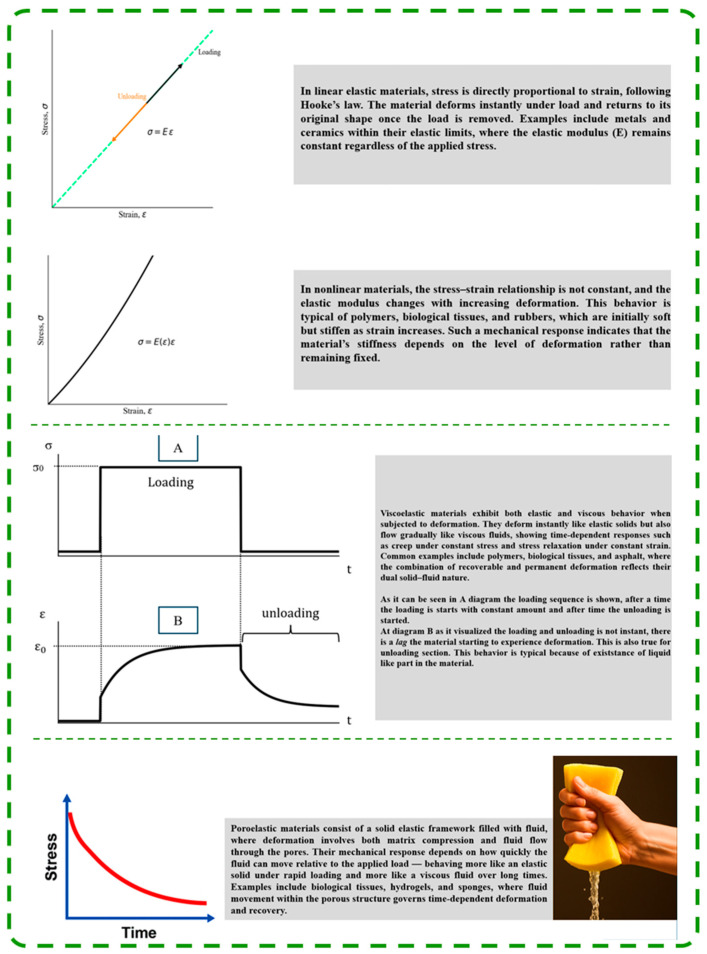
The different types of typical mechanical behavior of materials.

The mechanical response of a biomaterial as shown in Figure 1, can be divided into two broad categories: elastic (time-independent) and viscoelastic (time-dependent).

**Figure 2:**
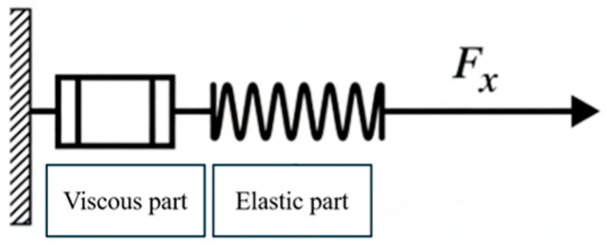
The Maxwell model for a typical biomaterial behavior. Note that there are two distinct parts: one spring represents elastic part of behavior, and rectangle shows viscous fluid-like one.

When a material such as a hydrogel is deformed, part of the stress is stored elastically while another portion gradually dissipates as the material relaxes. This stress relaxation can be described using the generalized Maxwell’s model (Figure 2).

$$G(t)=G_{\infty }+\Sigma G_{i}e^{-t/\tau_{i}}$$

Here, $G_{\infty }$ represents the equilibrium modulus (the remaining stiffness after full relaxation), while $G_{i}$ and *τi* represent the modulus and relaxation time of each molecular relaxation process. Short relaxation times reflect quick reverse deformation, whereas long ones indicate slower structural responses [106,107].

When these materials are loaded, the response is somewhat like gels, which have resonant deformation until full relaxation occurs. Under that oscillatory stress, a material’s total response can be expressed as:

$$G^{*}(\omega )=G^{\prime }(\omega )+iG^{\prime \prime }(\omega )$$

where *G′* is the storage modulus (elastic component) and *G″* is the loss modulus (viscous component). Their ratio, tan *δ = G″/G*′, shows whether the material behaves more like a solid or a liquid. When tan δ is small, the material behaves elastically, while higher values indicate viscous or fluid-like behavior. In hydrated materials like hydrogels, poroelasticity is the ability of water to flow through the matrix, which adds another layer of complexity. Because fluids are incompressible, this flow contributes to more stiffness for the material. The total stress tensor for these materials can be represented as:

$$\sigma_{ij}=2G\varepsilon_{ij}+\lambda \varepsilon_{kk}\delta_{ij}-\alpha p\delta_{ij}$$

where *G* and *λ* are Lamé constants, ε*_ij_* is strain, *p* is pore pressure, and *α* links fluid pressure to solid deformation. The poroelastic contribution varies with material porosity and permeability. One good example of this behavior is the sponges under water flow. If a sponge is compressed, one can feel the pressure in one’s hand because of the existence of the water fluid inside the sponge.

### 3.2. Mechanotransduction: Translating Force into Cellular Response

**Figure 3:**
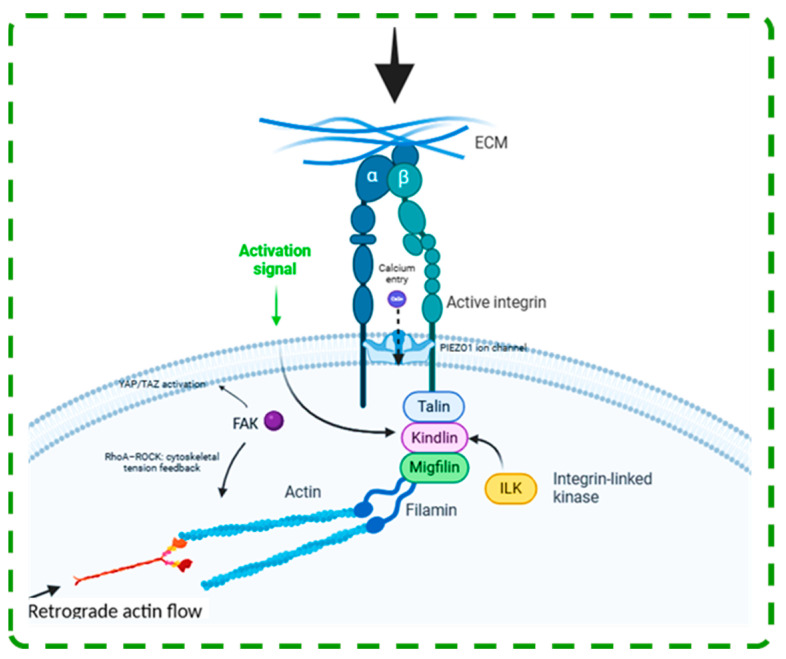
Simplified mechanotransduction map linking ECM mechanical inputs (stiffness, viscoelastic relaxation, and dynamic loading) to cellular sensors (integrins/focal adhesions and mechanosensitive ion channels), intracellular pathways (FAK/Src, RhoA/ROCK, Ca^2+^ signaling) [106,107,108].

Building on the mechanical descriptors introduced in Section 3.1, mechanotransduction refers to the cellular process of converting ECM mechanics into biochemical signaling and gene expression programs. In most tissues, this conversion is dominated by integrin-mediated focal adhesions (FAs) that physically couple the ECM to the actin cytoskeleton, enabling cells to probe whether their environment is compliant or stiff, dissipative or elastic, and static or time-varying [106] (Figure 3).

Integrins bind extracellular matrix ligands to form focal adhesions (FAs) that act as both mechanical anchors and signaling hubs. Force-dependent FAs regulate the activation of key components, such as talin, vinculin, and associated kinases, enabling cells to convert nanoscale binding events into traction forces at the cell matrix interface [106]. Consequently, cellular perception of mechanosensitive biomaterials depends not only on bulk stiffness but also on interfacial features such as ligand presentation and tether mobility, which critically shape cell hydrogel interactions. Actomyosin contractility generates intracellular tension that is transmitted to the matrix through the FA molecular clutch. The ability to sustain traction is strongly governed by the timescale of matrix mechanics: rapid stress relaxation can prevent stable FA maturation, whereas excessively slow relaxation or highly elastic matrices may impose resistance that limits cell spreading and remodeling [107]. Mechanical signals transmitted through FAs activate pathways such as FAK/Src and RhoA/ROCK, which regulate cytoskeletal organization, contractility, and downstream gene expression. Cellular responses are therefore more accurately interpreted by considering how stiffness, viscoelasticity, and nonlinear mechanical behavior collectively support traction forces, rather than stiffness alone. In parallel, mechanosensitive ion channels, including PIEZO1 and TRPV4, respond to membrane deformation by generating rapid calcium signals that modulate cytoskeletal dynamics and matrix remodeling [108]. These fast pathways interact with slower adhesion-mediated mechanisms to produce integrated outcomes such as changes in migration, differentiation, and extracellular matrix organization that can further reshape local matrix mechanics, particularly in three-dimensional environments. As a result, mechanobiological behavior is best understood through the combined effects of loading mode, temporal dynamics, and matrix adaptability, rather than any single mechanical parameter in isolation.

### 3.3. Mechanobiology-Guided Scaffold Design

This section discusses how scaffold mechanical properties influence cell behavior, with emphasis on traction support, relaxation dynamics, and nonlinear mechanical responses. The focus here is narrower and mechanobiology-driven: we highlight studies that explicitly connect a controllable mechanical feature of a scaffold (e.g., traction support, stress relaxation, nonlinear stiffening, or dynamic loading) to a mechanotransduction response and, ultimately, to a functional tissue outcome. This emphasis helps clarify what is actually being engineered when a scaffold is called “mechanosensitive.”

A first design question is whether the scaffold can support stable cellular traction. Cells do not respond to bulk stiffness alone; they respond to how forces are transmitted through focal adhesions, which depends on bulk mechanics and also on interfacial features such as ligand presentation and tether mobility. This is why scaffolds with similar elastic moduli can still produce different spreading, migration, and lineage trends when the interface or dissipation changes how traction is stabilized at focal adhesions. In practice, designs that decouple load-bearing structure from a more cell-instructive phase are often useful. For example, hybrid concepts pairing an elastomeric framework with a permissive, bioactive hydrogel phase allow mechanical responses like shape retention and load support without sacrificing cell-friendly porosity and factor interactions [108]. Related ideas are also seen in vascular morphogenesis studies, where matched initial stiffness did not guarantee the same outcome: endothelial programs such as integrin clustering and FAK activation proceeded only when the matrix mechanics supported the right traction dynamics, despite similar small strain G′ [108].

A second design question is how the mechanics of hydrogel are changed over time. Across many systems, time-dependent behavior often predicts outcomes better than a single static modulus. Early mechanobiology studies already showed that dynamic compression can improve cartilage-like tissue formation compared with static culture, and cyclic stretching can bias stem cells toward tenogenic programs, emphasizing that mechanical rhythm matters [106]. Moving from in vitro to in vivo, Xie et al. extended this idea using porous PLCL scaffolds in cartilage repair: rubber-like elasticity enabled repeated compression, and dynamic loading promoted more cartilage-like features than static conditions [107]. The practical message is that a scaffold’s utility is partly defined by how it behaves under the deformation cycles of the target tissue, not just by what it measures at rest. This principle is also consistent with large animal reconstruction results where macroscopic elastic recovery matched to the tissue environment was associated with improved integration and reduced adverse encapsulation, even when viscoelasticity was not the primary tuning target [109].

Within time dependence, stress relaxation deserves special attention because it directly controls whether cells can build and maintain traction during adhesion reinforcement. A major advance in this area came from studies that kept modulus and ligand density constant while changing relaxation behavior. Chaudhuri and co-workers showed that fast-relaxing networks promoted spreading and osteogenic commitment compared with more purely elastic gels, and linked these outcomes to integrin clustering and adhesion maturation [110]. Gong et al. then provided a simple mechanistic framing: spreading was maximized when the gel relaxation timescale matched the effective lifetime of cell matrix adhesions, and traction weakened when τ was far smaller or far larger than the cellular timescale [111]. This view turns τ into a design dial: it can be selected by choosing reversible bond chemistry, supramolecular unbinding kinetics, or network density so that yielding occurs on a biologically relevant timescale [110,111]. Importantly, additional work argues that stress relaxation can act as a primary cue for spreading independent of initial stiffness, reinforcing the need to report relaxation behavior and loss modulus rather than G′ alone [112]. The main tradeoff is practical: very fast relaxation can support remodeling and spreading, but it may compromise shape fidelity or load-bearing performance under physiological forces, so τ should be chosen in the context of the target tissue and the intended cellular process (adhesion, migration, differentiation).

Nonlinear mechanics is another recurring theme because many tissues experience large or repetitive deformations. Strain stiffening or strain softening changes what cells experience when forces increase, and it can provide compliance when needed and protection when necessary. Dynamic covalent networks have been used to achieve this balance: for example, strain-stiffening yet self-healing PEG-based systems combined toughness under larger deformations with permissiveness at low strain, and the reported links between nonlinear elasticity and bond exchange/topology provide actionable routes to tune the onset and slope of K′(γ) [113]. Follow-on mechanistic work highlighted that the same dynamic chemistry can either stiffen or dissipate depending on kinetics and network architecture, and it emphasized reporting differential modulus across strain and hysteresis across repeated cycles (since one-shot curves can be misleading) [112]. This caution is reinforced by analyses noting that dissipative Mullins effects may mask true stiffening unless protocols include repeat loading, and by arguments for testing nonlinear behavior under physiologically inspired histories rather than a single monotonic ramp [110]. Fiber-based strategies provide a complementary route: self-assembled semi-flexible networks can reproduce ECM-like nonlinear elasticity and then be chemically fixed, preserving processability while producing matrices that remain compliant at low strain and stiffen under larger deformations, useful when clinical robustness and cell permissiveness must coexist [114]. The tradeoff here is again context-dependent: if nonlinear reinforcement “turns on” too early, cell-driven remodeling and migration can be restricted; if it turns on too late, the construct may fail to protect function during handling, rehabilitation, or in vivo loading [112,115].

Beyond passive properties, a growing set of studies treats scaffolds as systems that can deliver controlled mechanical “doses” through actuation or energy transduction. The concept is that small, well-timed mechanical signals can bias mechanotransduction pathways without needing bulky hardware. Ozkale et al. articulated this direction by framing scaffolds as platforms that can sense inputs and generate outputs aligned with pathways such as integrin–FAK/Src, YAP/TAZ, or mechanosensitive channels (PIEZO1, TRPV4) [116]. Several examples follow this logic in different ways. Self-powered patches can convert body motion into microcurrents that stimulate PIEZO1-linked mechanotransduction and enhance angiogenesis and wound closure without external batteries [117]. Temperature-responsive adhesive hydrogels can contract at body temperature to physically close wounds and influence mechanosignaling (including YAP-related pathways), showing that gentle, physiologically driven actuation can be therapeutic [118]. Remote stimulation can also be layered onto structural scaffolds: magnetically guided mechanoactive mineralization scaffolds translate modest fields into microscale deformation, activate PIEZO1→β-catenin/YAP signaling, and accelerate osteogenesis and angiogenesis in vivo [103]. A related tissue-specific strategy is seen in tendon mimetic membranes combining aligned piezoelectric components with structure: normal motion generates small currents, reduces inflammation, and supports aligned collagen restoration, effectively coupling architecture with timing [119].

An important extension of “mechanical dosing” is that cells can respond to the frequency content of mechanical signals, not just their average magnitude. Phototunable hydrogels make this especially clear because stiffness can be changed rapidly and reversibly during culture. Oscillatory protocols showed that cells can produce stronger long-term traction at particular stimulation frequencies, supporting the idea that mechanosensing has its own timing rules and that frequency can be a design variable [120]. This also helps interpret why some dynamic regimens work while others do not: signals delivered at biologically relevant rhythms can accumulate, while very fast pulses may be averaged out.

Finally, the field is moving toward clearer “design maps” and reporting standards so that results can be compared across labs and translated into engineering rules. Frameworks have proposed a compact set of mechanical dials: instantaneous modulus, relaxation half-time, nonlinear stiffening, and plasticity, and linked them to measurable biological readouts such as integrin clustering, FAK/Src activity, MMP expression, YAP/TAZ localization, and PIEZO/TRPV4 signaling [121]. A recent methods and standards review similarly emphasized protocols for relaxation spectra, frequency-dependent moduli, nonlinear elasticity, and aligned cell readouts, aiming to make mechanobiology experiments more reproducible and portable [122]. From a design standpoint, these efforts address a central limitation of earlier literature: many studies reported only a single modulus, even though cells respond to a combination of traction support, time dependence, nonlinearity, and loading history.

Taken together, current evidence supports a simple but practical view: (i) traction support is a joint function of interface and bulk mechanics; (ii) relaxation and dynamic loading set “when” mechanics is sensed and can dominate outcomes even at equal stiffness; (iii) nonlinear behavior and hysteresis control performance under larger strains and repeated cycles; and (iv) actuation strategies expand scaffold design from choosing a modulus to programming a mechanical signal in time [106,107,108,109,110,111,112,113,114,115,116,117,118,119,120,121,122,123,124,125]. Key open questions remain, especially for translation: how to decouple mechanics from concurrent changes in porosity, ligand density, and degradation in 3D systems; how to guarantee long term stability of relaxation and nonlinear properties under remodeling and fatigue; and how to standardize “mechanical dose” (magnitude, frequency, waveform) in a way that is both biologically meaningful and manufacturable at scale [121,122].

## 4. Bioelectronic and Electroactive Hydrogels for Tissue Engineering

A variety of electroconductive dopants, such as carbon-based materials (nanotubes, graphene), metallic nanoparticles (gold, silver), and polymers (polyaniline, polypyrrole, polythiophene, and their derivatives), have been incorporated into hydrogels that display properties akin to biological tissues. These hydrogels have been designed to display certain attributes. Hydrogels of this kind have been engineered to enhance their mechanical and electroconductive properties [111,112].

Cell treatment and tissue-engineered cardiac patches are two potential therapeutic uses of electroconductive hydrogels in combating cardiovascular disease [109,116,117,123]. The objectives of these methodologies are to enhance muscle regeneration, diminish cardiac fibrosis and rigidity, and reinstate contractile function [108,113,115,118,120]. Transparent polymers, graphene oxide (GO), bionic liquids, nanomaterials (including carbon nanotubes [CNTs] and metallic nanoparticles), and other electroconductive materials might enhance the electroconductivity of hydrogels and rehabilitate the heart’s contractile function [110,114,119,121,122,124,125,126,127,128,129,130]. Shin et al. increased gelatin methacryloyl (GelMA) hydrogels by including sheets of reduced graphene oxide (rGO). The impedance values were reduced via rGO. Hybrid hydrogels including rGO demonstrated significantly decreased impedance (*4 kΩ) at a concentration of 1 mg/mL, in contrast to hydrogels formulated with nonreduced GO (*120 kΩ) [131]. Wang et al. administered hydrogels composed of HA/PEG/tetraaniline, infused with stem cells derived from adipose tissue, into the myocardium of rats who had a myocardial infarction [121]. This therapy reduced fibrosis at the site of the infarct and restored cardiac output. This study addressed a significant issue with practical implications: the lack of vascularization in transplanted cells or tissues [113,115].

Conductive polymers are a category of conjugated polymers characterized by a chain backbone consisting of alternating single and double bonds. The polymers include polyacetylene, polypyrrole (PPy), polythiophene, poly(3,4-ethylenedioxythiophene) (PEDOT), and polyaniline (PANi) [132,133]. The aromaticity of the chain backbone facilitates electron transfer between adjacent chains. Conductive polymers used in electroactive tissue engineering have a conductivity comparable to that of semiconductors, ranging from 10^−3^ to 10^5^ S cm^−1^. Liang and colleagues produced a pyrrole monomer-capped hyperbranched polymer, referred to as hyperbranched poly (amino ester) (HPAE)-Py. This polymer may be used for treating cardiac problems by facilitating concurrent gelation and conductivity generation in situ in the presence of Fe^3+^. In accordance with the electronic percolation concept, these hydrogels demonstrate a significant conductivity of 10^−4^ S cm^−1^ when present at substantial concentrations of HPAE-Py, namely between 30% and 50% [114]. Xu and colleagues did a distinct experiment, opting for heparin as a substitute for polystyrene sulfonate (PSS) to manufacture PEDOT: heparin. It was incorporated into a dynamic hydrogel matrix to create injectable, bio-adhesive ECH. This procedure was comparable to the evolution of the traditional conductive polymer PEDOT:PSS [134].

Electroconductive hydrogels have the potential to be used in the regeneration and repair of neurological tissue, the enhancement of endogenous cell signaling, and the transmission of electrical stimulation from the outside [135]. An illustration of this would be the regeneration potential of gels containing chitosan and coated with PEDOT [136]. A recent study conducted by Liu and colleagues showed that the use of the hydrogen bond donor poly(2-(methacryloyloxy)ethyl)trimethylammonium chloride in the synthesis of electroconductive hydrogels resulted in a considerable increase in the number of cells that had neurites [137]. These hydrogels have the potential to stimulate neurite outgrowth and stimulate Schwann cells to produce higher quantities of neurotrophic factors. Neurotrophic factors are essential for the survival, development, and functioning of neurons [138].

Lee et al. investigated the many functions and developmental stages of synthetic cardiac tissue by electrophysiological investigation and cardiac cell activity. The tissue was produced using three distinct hydrogel scaffolds composed of gelatin methacrylate (GelMA) and including various carbon nanomaterials (CNT, GO, reduced graphene oxide, or rGO). Direct evidence indicates that the electroconductivity properties of ECH scaffolds significantly influence seed cell behavior, as CNT and rGO-infused GelMA conductive hydrogel scaffolds demonstrated superior organization and maturation of cardiac cells compared to GO-infused GelMA nonconductive hydrogel scaffolds [139].

As a result of thermodynamic diffusion and random collisions between nanoparticles, hydrogels enhanced with noble-metal nanocrystals (such as Au, Ag, or Pt) exhibit higher electrical properties. The method devised by Baei et al. included the simultaneous synthesis of Au nanoparticles and gelation upon injection into defective areas; this allowed the Au nanoparticles to maintain a uniform dispersion inside the hydrogel network. All things considered, electroconductive fillers made of noble metal nanocrystals have great conductivity, persistent chemical inertia, and excellent biocompatibility, making them ideal for use in tissue engineering scaffolds for regeneration, particularly in the restoration of heart tissue [140].

Zhou and colleagues synthesized a biocompatible, soft, and highly conductive electrochemical hyde (ECH) using tannic acid (TA), polypyrrole (PPy), and Fe^3+^. In vitro, the ECH suppressed astrocyte proliferation and promoted the differentiation of neural stem cells (NSCs) capable of generating neurons [141]. Moreover, Xu et al. used germanium phosphide (GeP) nanosheets, which are biodegradable and electrically conductive, to include hyaluronic acid, grafts, and dopamine hydrogel. Moreover, in vitro, ECHs possess the capability to expedite the differentiation of neural stem cells into neurons [142].

In reaction to electrical impulses, the skin displays conductivity ranging from 2.6 to 1 × 10^−4^ mS cm^−1^, just as the heart and nerves. A two-dimensional bio-nanosheet made of cellulose crystal and PDA-reduced-GO was designed and manufactured by Yan et al., and it is conductive. Biostability, conductivity, flexibility, and exceptional cell and tissue affinity performance were all displayed by the hydrogel made of nanosheets. When used in conjunction with the appropriate electrical stimulation therapy, conductive hydrogels effectively change cell shape, proliferation, elongation, and differentiation. The ES groups showed enhanced angiogenesis and faster wound healing in a diabetic mouse dermal wound model [143]. Furthermore, conductive biomaterials and electroconductive hydrogels have garnered significant attention among tissue engineering researchers. Recent developments in electroconductive hydrogels for tissue engineering applications are summarized in Table 2.

## 5. Advanced Biofabrication and Programmable Architectures

Cells don’t experience biomaterials as static plastic shapes; they experience time-varying mechanics and evolving architectures. This section zooms out one level: how those materials are actually fabricated as three-dimensional (3D) and four-dimensional (4D) constructs, and how they can be delivered directly into defects or onto tissues via in situ bioprinting and minimally invasive printing. The core idea is simple: fabrication and deployment must preserve (or deliberately program) the dynamic mechanical features that cells read, relaxation, nonlinear stiffening, and actuation, rather than erasing them with rough processing [155,156,157].

### 5.1. Design Constraints for 3D Printable Adaptive Biomaterials

To be printable, an adaptive bioink has to satisfy two competing demands. On the printer, it must behave like a viscoplastic ink: shear thinning under high shear in the nozzle, holding shape after deposition with a finite yield stress. In culture or in vivo, the same material must relax, remodel, and transmit biologically relevant mechanical inputs. Most 3D/4D inks, therefore, rely on a dual network strategy in which a fast, reversible printing network sits on top of a slower, mechanobiologically relevant network [155,158,159]. For extrusion-based biofabrication, the printable regime is roughly defined by a storage modulus high enough to avoid collapse at body temperature and strong shear thinning. Microgel-based inks and reversible supramolecular networks are widely used as the sacrificial or secondary network, because they are strong enough to hold filaments but weak enough that they fluidize under shear and heal within seconds after deposition [155,158]. The mechanoadaptive behavior is then encoded in a second network: a covalent or dynamic covalent matrix with tunable stress relaxation and nonlinear elasticity. Classic examples include alginate or PEG networks with controlled ionic or dynamic covalent crosslinks that reproduce the stress-relaxing behavior of natural ECM and support mechanosensitive spreading, proliferation, and osteogenic differentiation of stem cells [160,161,162]. When the material’s relaxation time aligns with the timescales of processes like focal adhesion and cytoskeletal remodeling, cells are able to spread far more effectively. So, the relaxation time can be a useful tool for controlling the ECM/Cell interactions [162]. Dynamic covalent chemistries like boronate esters, Schiff bases, Diels–Alder, and disulfides are especially attractive because they combine mechanical stability with bond exchange. Hydrogels based on these bonds can show solid-like behavior under quasi-static loading, yet relax stress, self-heal after damage, and be reprocessed or printed [163]. One PEG network crosslinked by reversible covalent bonds exhibited strain stiffening under large deformations but recovered its integrity after unloading, combining the stiffness needed in the surgery and fabrication with softness needed in the actual cell’s environment [164]. Topology is the other hidden variable. Work on semi-flexible fiber networks has shown that strain-stiffening emerges when fibers are long, marginally connected, and able to reorient under load. Hydrogels that mimic the fibrous architecture of collagen by self-assembly followed by covalent fixation reproduce this nonlinear elasticity while remaining printable, offering a route to ECM-like mechanics in 3D printed constructs [165]. In practice, a 3D-printable adaptive biomaterial ends up being a compromise: the composite ink relies on a sacrificial microstructure to control its rheology during printing, while the long-term cellular response is governed by a slower-forming, weaker crosslinked or fibrillar network that ultimately directs the mechanobiology.

### 5.2. 3D Printing Strategies for Mechanosensitive Constructs

Extrusion-based bioprinting (direct ink writing) is the workhorse for adaptive hydrogels. It tolerates high viscosities, multimaterial cartridges, and cell-laden formulations. Gradients in stiffness, relaxation time, or bioactive ligand density can be encoded either along the print path (by switching inks) or across filament cross-sections using coaxial and multichannel nozzles. A particularly powerful variation is 3D microfluidic assisted spinning (3DMB), in which multiple streams of prepolymer, crosslinker, and fillers are combined in a microfluidic head before extrusion. This generates filaments with controlled core-shell structures, anisotropic fillers, and longitudinal gradients in composition that would be impossible with a simple syringe nozzle [157]. Because microfluidics can precisely control flow rates and diffusion distances, it becomes possible to tune the spatial distribution of dynamic crosslinkers, nanoparticles, or mechanosensitive domains along and across each printed fiber. For example, a filament might have a softer, fast-relaxing core to encourage cell infiltration and a stiffer, strain-stiffening shell to handle surgical loads. In a layered scaffold, this patterning method enables spatially programmable mechanotransduction behavior. In this approach, cells at the interface experience one mechanical response while cells deeper in the construct experience another. Inkjet and laser-assisted techniques trade viscosity tolerance for resolution. They are better suited for patterning low viscosity bioinks, growth factors, or adhesive motifs onto preformed adaptive scaffolds rather than building the whole structure from scratch. These methods can deposit microliter droplets of crosslinker, ligand, or nanoparticle modifiers that locally alter relaxation time, nonlinear stiffening, or degradation without changing the scaffold’s bulk geometry [155]. Vat photopolymerization (DLP, twophoton) excels at generating complex architectures with micrometer scale resolution, but historically has struggled with overcrosslinking networks into purely elastic, brittle glasses. Recent work has addressed this by using photo-triggered dynamic chemistries such as photolabile crosslinkers, photo-induced bond exchange, or secondary networks that can be softened or stiffened after printing [159]. This allows stiff load-bearing lattices to be printed and then locally relaxed or softened to restore cell-permissive viscoelasticity. In principle, this means one can print a corneal, cartilage, or ligament mimetic framework whose subregions are tuned post-fabrication to match the local mechanical microenvironment.

### 5.3. 4D Biofabrication: Printing Time-Programmed Mechanics and Shape

**Figure 4:**
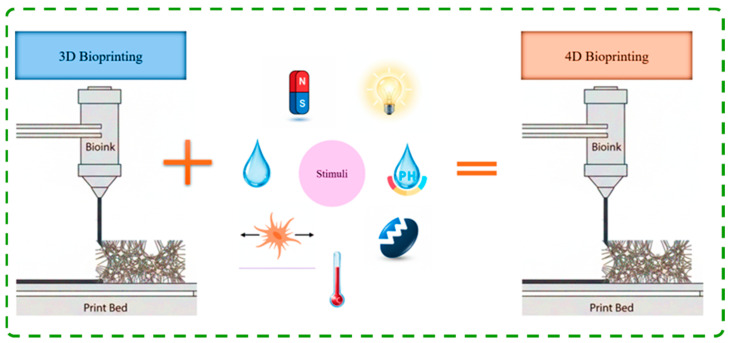
4D printing comprised from a 3D printing mixed with one or more stimuli, which results in dynamic printed polymer.

4D printing extends standard 3D printing by adding time as an explicit design axis (Figure 4). In these constructs, geometry, stiffness, or porosity evolve predictably after printing in response to stimuli such as water, temperature, pH, enzymatic activity, light, magnetic fields, or even cell traction [156,166]. From a mechanobiology perspective, 4D printing is valuable because it allows us to program when and how mechanical signals appear. One important strategy is swelling-driven shape morphing. Hydrogels with anisotropic swelling (imposed by print path, filler alignment, or layered compositions) can fold, curl, or twist when hydrated or when ionic conditions change. By aligning print strands or gradients, one can design constructs that gently close over a defect, change curvature to match surrounding tissue, or open internal channels as matrix degrades [155]. A second common approach uses thermo- and moisture-responsive shape memory polymers. These materials are printed in a temporary shape that can later recover a memorized configuration at body temperature or upon hydration. In tissue contexts, this allows minimally invasive insertion of a compact device that then expands or bends into a functional shape, delivering controlled mechanical strain or pre-stress to surrounding tissues [156].

A third route relies on dynamic covalent and supramolecular networks. Covalent hydrogels are practically 4D because their mechanical properties evolve as bonds exchange. Systems based on reversible covalent crosslinks or combined covalent/supramolecular networks can stiffen under load (strain stiffening) while recovering when load is removed, or progressively soften as network rearrangement accumulates [163,164,165]. When these are printed into non-uniform architectures, the time-dependent mechanics become spatially heterogeneous: edges may stiffen faster under eye blinking or joint motion, while cores remain soft. Finally, in many systems, cells themselves provide the fourth dimension. As they contract, remodel ECM, and deposit new matrix, they cause printed sheets or capsules to fold, buckle, or compact (cell origami) [156]. In adaptive scaffolds, this behavior is not a failure mode but a design feature: tissues are allowed to self-organize into curved or layered structures that better resemble native anatomy. The key lesson from mechanobiology is that 4D behavior should not just be about shape aesthetics. If the time-course of stiffening, softening, or actuation is matched to cellular mechano-sensing timescales like minutes to hours for focal adhesion maturation, days for ECM deposition. Cells integrate the dynamic cues and change fate more robustly than on static analogues. Stress-relaxing hydrogels and networks with controlled viscoelastic spectra have already shown that faster relaxation enhances spreading, proliferation, osteogenesis, and even vascular morphogenesis by permitting integrin clustering and FAK activation while dissipating excess stress [160,162]. Incorporating these same principles into 4D printed constructs converts shape change into mechanobiologically meaningful work. On the more active side, mechanoactive scaffolds that convert external magnetic fields into micro deformations have been proposed for bone repair. They essentially act as remote-controlled 4D systems: applying low-amplitude, programmable strains along with biochemical cues, and activating mechanosensitive pathways such as PIEZO1–YAP/β-catenin to accelerate osteogenesis and angiogenesis [105]. These platforms show how 4D printing, mechanosensitive materials, and non-invasive actuation can be combined into fully programmable therapeutic devices.

### 5.4. In Situ Bioprinting and Minimally Invasive Deployment

Conventional tissue engineering follows an “ex vivo then implant” model: fabricate a construct in the lab, mature it, then surgically implant it. In situ bioprinting flips this logic by fabricating directly on or within the defect, using patient anatomy as the build platform. Handheld and robotic in situ bioprinters for skin already exist in preclinical and early translational studies. These devices deposit hydrogel-based bioinks (cell-laden or cell-free) directly onto burns and wounds, with real-time motion compensation and simple crosslinking schemes (for example, ionic or light-triggered systems) [158]. For adaptive materials, in situ deployment adds several important layers. First, geometric conformity is achieved without over-engineering the macro geometry. Because the scaffold is printed directly onto the tissue, its shape is automatically patient-specific, so design effort can be focused on internal architecture and mechanical behavior rather than design. This is particularly relevant in regions where curvature, layer thickness, or defect depth are hard to capture ex vivo. Second, multi-chamber cartridges allow on-demand tuning of mechanical properties in real time. Surgeons can modulate composition on the fly, printing a more elastic, fast-relaxing ink in regions that must undergo large deformations and a stiffer, slower-relaxing ink along load-bearing edges, with appropriate rheology enabling such switches within a single continuous print. Third, in situ printing improves integration with native mechanics. Viscoelastic hydrogels designed for nucleus pulposus or fibrocartilage regeneration have shown that matching relaxation behavior and ion channel activation (TRPV4, PIEZO1) to the native tissue can preserve phenotype under complex loading [6,7,8,13]. Printing such materials directly into a disc or joint defect means the construct experiences physiological loading from day one, rather than an artificial in vitro regimen. Finally, minimally invasive 3D printing (MI3DP) concepts extend this approach into deeper tissues. Here, the print head is inserted through keyhole incisions or natural orifices and constructs the scaffold in situ within confined defects. In bone and cartilage, this enables filling of deep, irregular cavities that would otherwise require open surgery. Mechanoadaptive materials are particularly attractive in this setting: they can be printed in a compact, flowable form and then expand, stiffen, or reorganize mechanically after deployment to provide support while still allowing stress relaxation and remodeling. Skin is the most obvious target, and recent work suggests that in situ printing could replace or augment split-thickness grafts by combining fast wound coverage with programmable mechanics and bioactive content [158]. The same logic extends to corneal patches, conjunctival reconstruction, joint cartilage defects, and disc annulus repairs, where curvature, transparency, or load-bearing all depend on fine-tuned mechanics that are difficult to reproduce with pre-formed implants.

### 5.5. Outlook: Integrating Fabrication, Mechanics, and Clinical Reality

The emerging picture is that 3D/4D biofabrication and in situ deployment are not separate topics from mechanobiology-informed material design; they are the implementation details that will decide whether mechanoadaptive scaffolds ever matter in patients. Current reviews on 3D/4D printing make it clear that additive manufacturing can already deliver complex geometries, multi-material constructs, and even time-programmed shape changes [155,156]. In parallel, mechanobiology has quantified how relaxation spectra, nonlinear elasticity, and network dynamics control spreading, differentiation, and vascularization [160]. The next step is to make those mechanical dials directly addressable in the printer. This implies print paths and fill patterns that deliberately encode anisotropy and strain stiffening along desired directions rather than treating these as incidental side effects. It also implies cartridge design and slicing algorithms that let users assign relaxation time and loss modulus, not just Young’s modulus, so that viscoelastic spectra become design targets [162]. Optimization of printing should include mechanosensing readouts such as integrin clustering, FAK and YAP activation, or ion-channel signaling. So that printability and bioactivity are cooptimized rather than traded off. On the translational side, compact in situ printers need to be engineered for realistic operating room conditions. They must be sterile, robust, and fast, relying on chemistries that crosslink under clinically acceptable light or ionic conditions. As these pieces come together, biofabrication stops being simply a way to draw shapes and becomes a way to sculpt the mechanical and temporal microenvironment that cells experience in vivo. Adaptive scaffolds printed and deployed this way have the potential not just to fill defects, but to actively choreograph regeneration [167,168].

## 6. Conclusions and Future Perceptions

Among a lot of research on materials and biomaterials for bioengineering and tissue repair, stimuli-responsive hydrogels as smart and adaptable biomaterials have been widely used in the field of tissue engineering. These smart biomaterials, due to their response to physical, chemical, and biological stimuli, have received extensive attention. In addition, they can also release biomolecules, drugs, genes, proteins, and various functional molecules, which greatly improves the therapeutic effect of disease. In this review, all of necessary details on responsive hydrogels, such as their classification, application, and function in different types of tissue repair. In conclusion, the advent of smart, adaptive hydrogels marks a transformative shift in tissue engineering, enabling dynamic interaction with biological environments to guide regeneration. Advances in mechanically responsive hydrogels, electroactive materials, and precision bioprinting have collectively expanded the potential for more effective, minimally invasive therapies that leverage the body’s own mechanical and electrical cues. Despite challenges in scalability, safety, and real-time validation, these innovations pave the way for personalized, information-rich implants that blur the line between living tissue and engineered constructs, heralding a new era of regenerative medicine.

In the future, developing smart and adaptable hydrogels for the next generation of tissue engineering is an omnipotent research area. They pave the way to develop a controllable responsive delivery platform for the biological environment and other disease microenvironments. In addition to the wide range of the moralities in smart-responsive hydrogel platforms, their responsiveness, biocompatibility, biodegradability, inflammation, and immune response are still challenges and limit their fabrication and functionalization process. Obviously, more attention should be paid to the field of smart-responsive hydrogels for next-generation tissue engineering in the future. For example, regarding their cytotoxicity risks, natural and biocompatible polymers, cross-linking agents, and biomaterials with hydrophilicity should be utilized to overcome the toxicity issues. However, when scientists overcome the above problems one by one in the foreseeable future, the structure and function of smart-responsive hydrogels will be continuously optimized. And tailored smart-hydrogel-based tissue repair strategies can be designed for different types of diseases.

## Figures and Tables

**Table 1:** Comparison of stimulus-responsive hydrogels, summarizing mechanisms, advantages, and key biomedical applications.

Stimulus Type	Mechanism	Advantages	Key Applications	Ref.
Ultrasound	Acoustic waves trigger hydrogel deformation, payload release, or electrical output	Non-invasive, controllable, self-healing, deep tissue penetration	Bone repair, nerve stimulation, anticancer therapy, tissue regeneration	[21]
Magnetic Field	Magnetic nanoparticles respond via motion, heat, or deformation	Remote control, precise spatiotemporal regulation, deep tissue penetration	Bone, cartilage, nerve, cardiac, skin tissue engineering; targeted drug delivery	[28]
Light (UV/Vis/NIR)	Photon absorption induces swelling, phase transition, or chemical modification	High spatial/temporal resolution, non-invasive, tunable	Tissue engineering, neuronal differentiation, antimicrobial dressings, bone repair	[44]
Temperature	Sol-gel transitions via hydrophilic/hydrophobic balance	Injectable, in situ formation, biocompatible, minimally invasive	Wound healing, bone/dental regeneration, soft robotics, pain management	[53]
Pressure/Mechanical	Compression or shear stress induces network deformation or drug release	Mechanically sensitive, wearable/injectable, localized delivery	Drug delivery under strain, vascular-targeted therapy, strain sensors, tissue engineering	[71]
pH	Protonation/deprotonation of polymer chains	Site-specific, responsive to acidic microenvironments	Chronic wound healing, bone regeneration, injectable scaffolds	[84]
Glucose	Glucose-binding (GOx, Con A, PBA) triggers hydrogel response	Self-regulated, dynamic, multifunctional	Diabetic wound healing, bone/cartilage repair, biosensors, 3D-printed scaffolds	[90]
ROS	Oxidative stress induces degradation or drug release	Reduces oxidative damage, immunomodulatory	Wound healing, spinal cord repair, cardiovascular therapy, stem cell delivery	[77]
Enzyme	Hydrogel cleaved or restructured by specific enzymes (MMPs, aggrecanase)	Tissue-specific, controlled degradation, high precision	Cartilage engineering, glioma therapy, tendon anti-adhesion scaffolds	[96]
Antigen/Antibody	Specific binding alters hydrogel network	Highly selective, sensitive, label-free	Biosensing, immunodetection, controlled drug delivery	[101]

**Table 2:** Electroconductive hydrogels for tissue engineering.

Hydrogel Component	Conductive Material	Application	Ref.
QCS-CD-AD/GO	GO	Wound healing	[144]
Aga/Gel/PPY (AGP3)	PPY	Spinal cord injury repair	[145]
Fmoc-K- (Fmoc)-RGDPANI	PANI	Cardiac tissue engineering	[146]
PNAGAPAMPS/PEDOT/PSS	PEDOT/PSS	Scaffold materials for soft tissue engineering	[147]
TA/PPY-Fe^3+^	PPY	Spinal cord injury repair	[148]
Hyaluronic acid	Cardiac Tetraaniline	Cardiac tissue engineering	[121]
PGS	Aniline	Neural tissue engineering	[138]
Alginate	CAGNF	Neural tissue engineering	[149]
GelMA or PEGDA	Bio-IL	Cardiac tissue engineering	[130]
PDA@rGO/cellulose nanofibers	PDA@rGO	Skin tissue engineering	[143]
polyHEMA/PPy	PPY	Diabetic wound	[150]
PEDOT-PDA-mSF	PEDOT	Diabetic wound	[151]
QCS-g-PANi/PEGS-FA	PANI	Antibacterial-wound healing	[152]
QCS/PDA@rGO/PNIPAm	GO	Tissue adhesive	[153]
PAA/TA-Ag nanozyme	TA-Ag	Infected wound healing	[154]

## Data Availability

No new data were created or analyzed in this study. Data sharing is not applicable to this article, as it is a review of previously published literature.
